# Supplementary material for: ATP-dependent membrane remodeling links EHD1 functions to endocytic recycling
Source: Nat Commun. 2018 Dec 5;9:5187. doi: 10.1038/s41467-018-07586-z (PMC6281616; doi:10.1038/s41467-018-07586-z)
Supplement: Supplementary file 1 — Supplementary Information [file 41467_2018_7586_MOESM1_ESM.pdf]

Supplementary Information

ATP-dependent membrane remodeling links EHD1 functions to endocytic recycling

Deo and Kushwah et al.

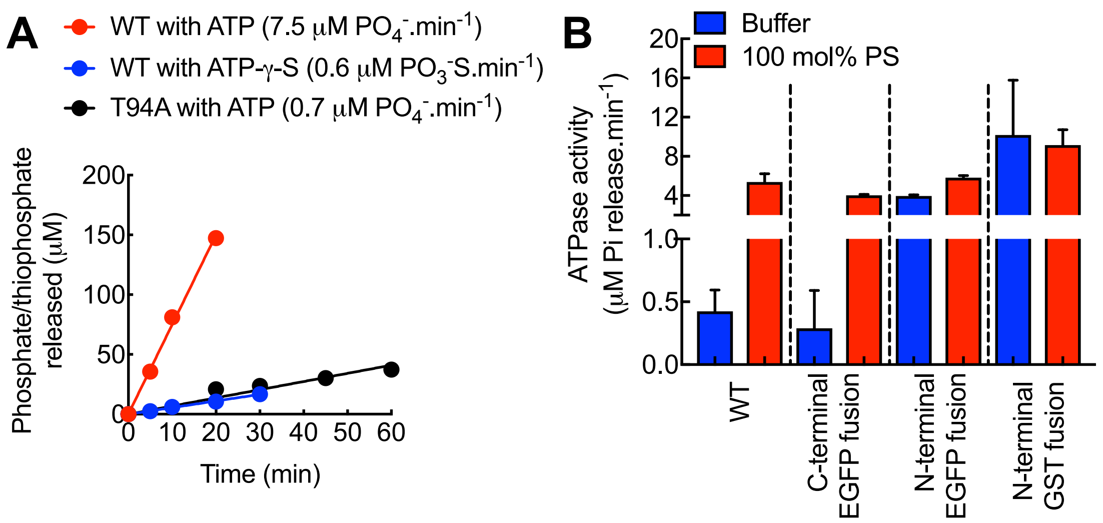

Supplementary Figure 1. ATPase activity of mutants and fluorescent constructs of EHD1. **(A)** Kinetics of nucleotide hydrolysis by EHD1 and EHD1(T94A). **(B)** ATPase activities of EHD1 constructs in the absence or presence of 100 mol% PS liposomes (mean ± SD, N = 3).

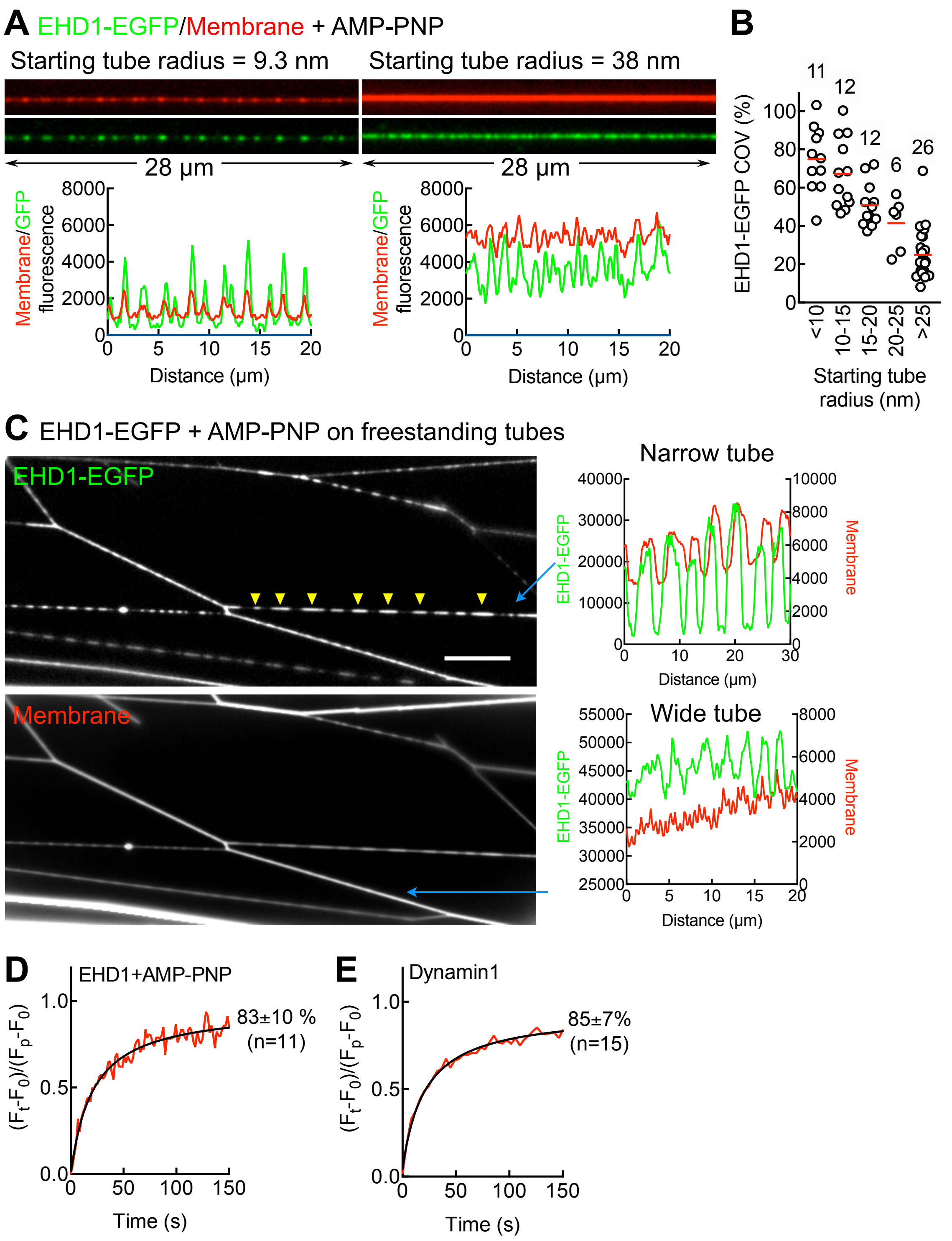

Supplementary Figure 2. Characteristics of EHD1 scaffolds. (A) Representative fluorescence micrographs and associated line profiles showing the distribution of EHD1-EGFP on tubes of the indicated sizes. **(B)** Coefficient of variation (COV) of EHD1-EGFP as a function of starting tube size for the indicated numbers of tubes. Red line denotes the mean. **(C)** Representative fluorescence micrographs and associated line profiles showing the distribution of EHD1-EGFP in

presence of AMP-PNP on freestanding tubes. Yellow arrowheads mark scaffolds of EHD1-EGFP on the tubes. Scale bar = 10 µm. Plots showing kinetics of fluorescence recovery after bleaching RhPE-containing tubes with AMP-PNP-bound EHD1 scaffolds **(D)** and dynamin scaffolds **(E)**. Numbers represent the mobile fraction (mean ± SD, n(tubes) as indicated).

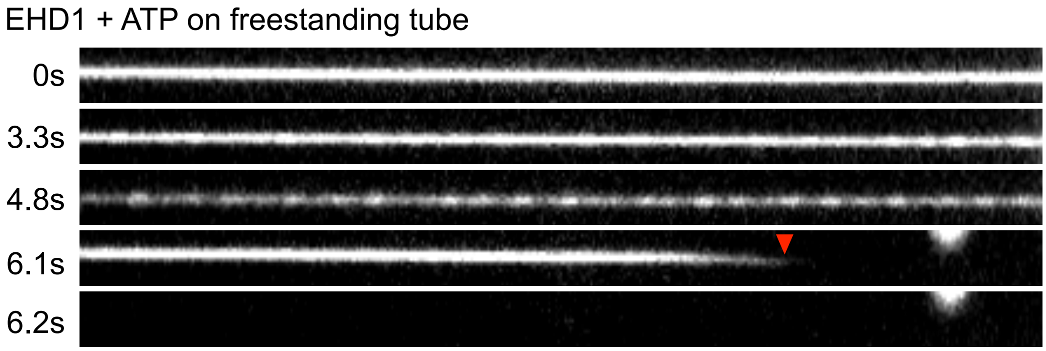

Supplementary Figure 3. EHD1-catalyzed fission on freestanding tubes. Frames from a time-lapse movie showing fission of freestanding tubes with EHD1 and ATP. Red arrowhead marks the site of scission.

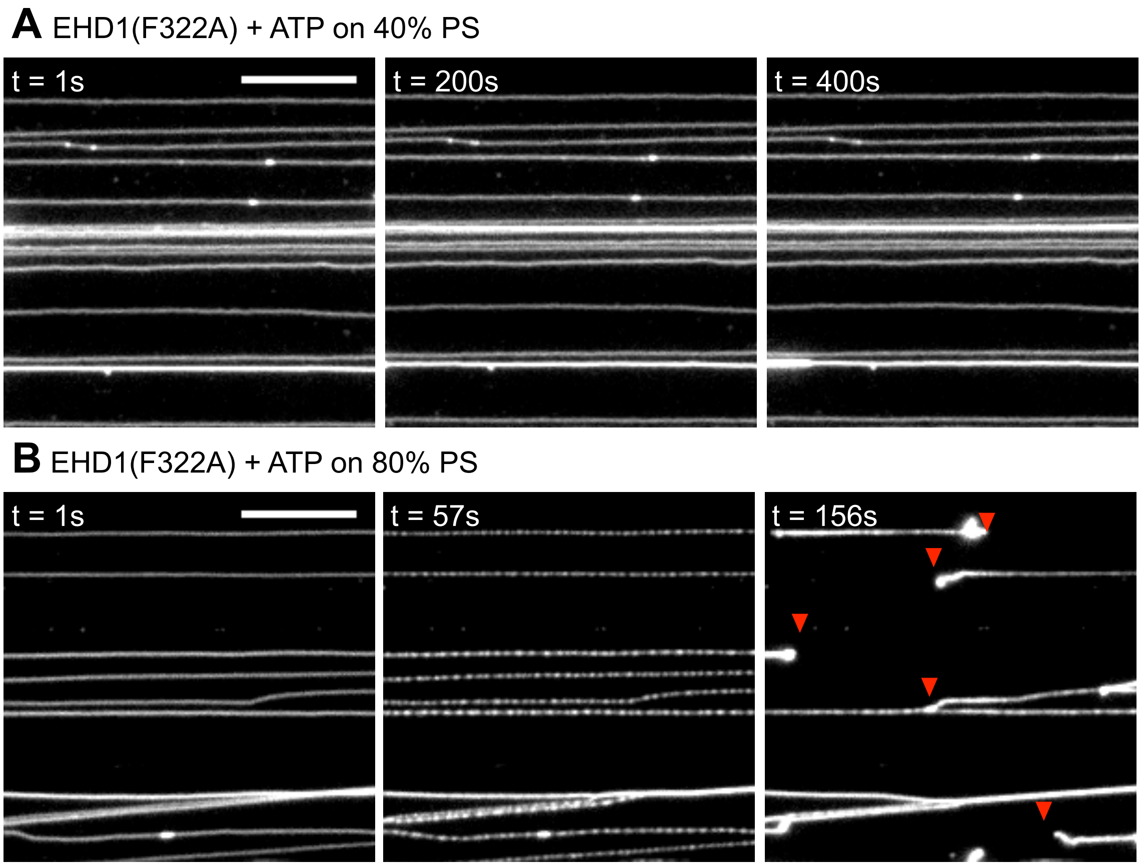

Supplementary Figure 4. Fission with membrane binding mutant of EHD1. Frames from a representative movie showing the effect of adding EHD1(F322A) with ATP on **(A)** 40 mol% and **(B)** 80 mol% PS templates. Red arrowheads mark cut ends of the tubes.

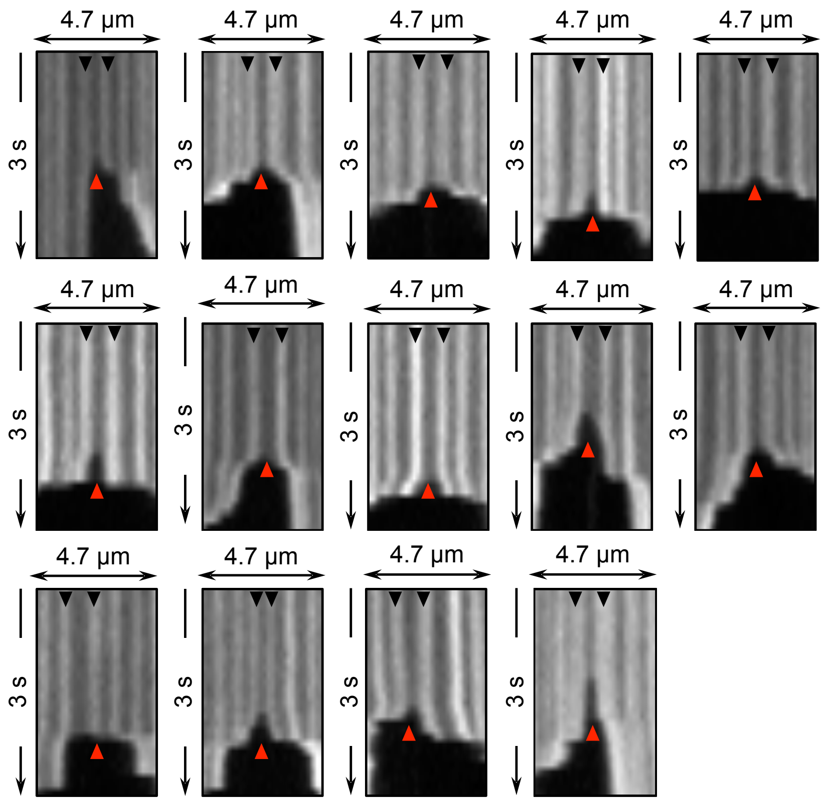

Supplementary Figure 5. Site of fission. Montage of kymographs showing that scission (red arrowheads) occurs between two bulges (black arrowheads) formed by EHD1 with ATP.

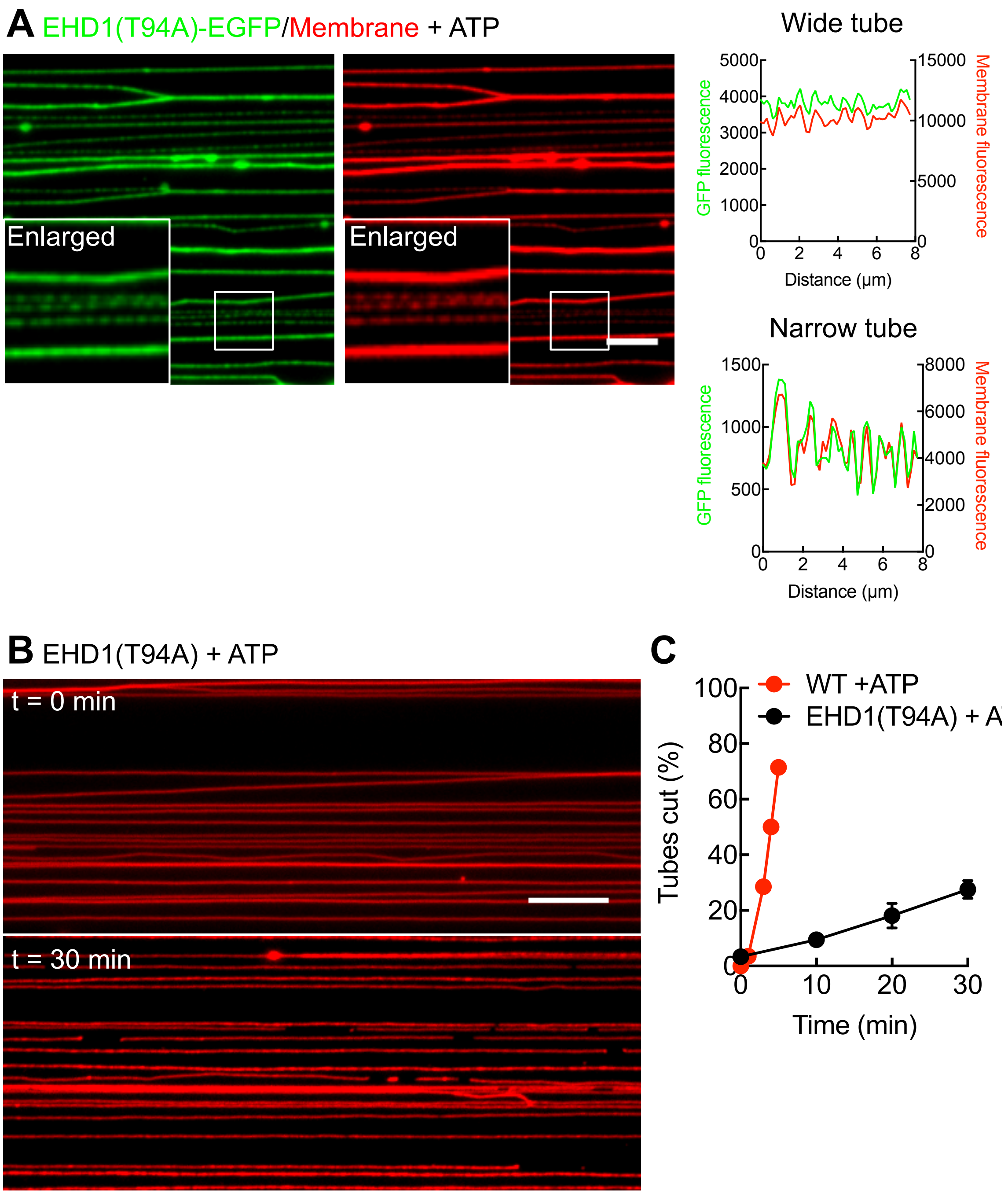

Supplementary Figure 6. Characteristics of the ATP hydrolysis-defective mutant of EHD1. **(A)** Representative fluorescence micrographs showing the distribution of EHD1(T94A)-EGFP with ATP on tubes. Scale bar = 5 μm. **(B)** Representative fluorescence micrographs showing the effect of adding EHD1(T94A) with ATP before and after 30 min. **(C)** Plot showing the kinetics of tubes cut (as percentage of the total tubes analyzed) with the indicated proteins. Data represent the mean ± SD.

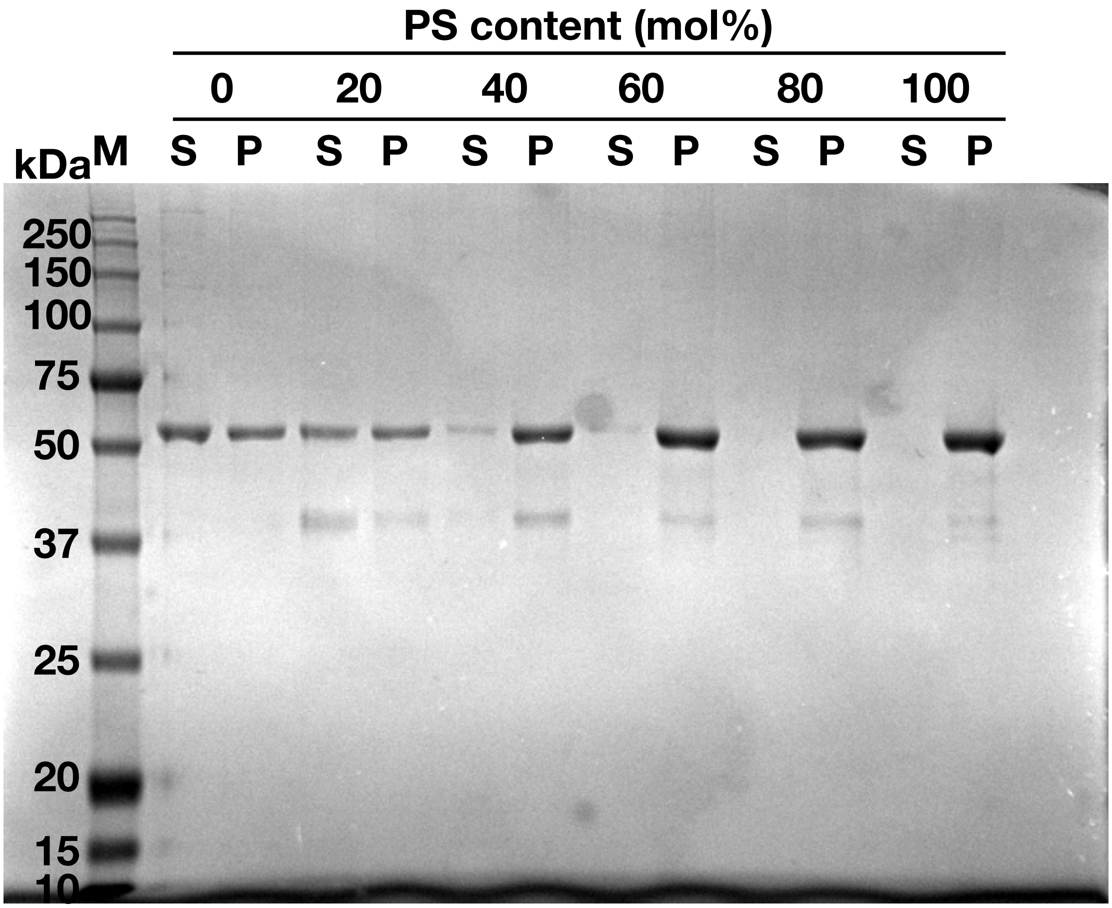

Supplementary Figure 7. Uncropped scand of an SDS-PAGE gel showing results from a liposome sedimentation assay. Lanes indicated are for the marker (M), supernatant (S) and pellet (P) fractions.
