# Supplementary material for: ATP-dependent membrane remodeling links EHD1 functions to endocytic recycling
Source: Nat Commun. 2018 Dec 5;9:5187. doi: 10.1038/s41467-018-07586-z (PMC6281616; doi:10.1038/s41467-018-07586-z)
Supplement: Supplementary file 3 — Description of Additional Supplementary Files [file 41467_2018_7586_MOESM3_ESM.pdf]

Description of Additional Supplementary Files

File Name: Supplementary Movie 1
Description: EHD1-induced membrane remodeling and fission. Movie showing the effect of adding EHD1 with ATP to tubes. Movie is acquired in the membrane fluorescence channel.

File Name: Supplementary Movie 2
Description: EHD1-induced membrane fission on freestanding tubes. Movie showing the effect of adding EHD1 with ATP to freestanding tubes. Movie is acquired in the membrane fluorescence channel.

File Name: Supplementary Movie 3
Description: Nucleation and growth of EHD1-EGFP oligomers with ATP. Movie showing the nucleation and growth of EHD1-EGFP oligomers on tubes.

File Name: Supplementary Movie 4
Description: Simulation of a 4 nm-long scaffold on a 100 nm-long tube. Magenta represents the scaffold. Cream and green spheres represent the headgroups of DOPS and DOPC, respectively. Light and dark spheres represent headgroups of lipids on the outer and inner leaflet, respectively. Only lipid head groups are shown for clarity.

File Name: Supplementary Movie 5
Description: Simulation of a 20 nm-long scaffold on a 100 nm-long tube. Magenta represents the scaffold. Cream and green spheres represent the headgroups of DOPS and DOPC, respectively. Light and dark spheres represent headgroups of lipids on the outer and inner leaflet, respectively. Only lipid head groups are shown for clarity.

File Name: Supplementary Movie 6
Description: Simulation showing the slice of the tube where fission takes place. Cream and green spheres represent the headgroups of DOPS and DOPC, respectively. Only lipid headgroups in the inner leaflet are shown for clarity.

File Name: Supplementary Movie 7
Description: Lack of effective membrane remodeling by EHD1(Δ2-9). Movie showing the effect of adding EHD1(Δ2-9) with ATP to tubes. Movie is acquired in the membrane fluorescence channel.
